# Morphology and microcirculation changes of the optic nerve head between simple high myopia and pathologic myopia

**DOI:** 10.1186/s12886-023-02949-7

**Published:** 2023-05-10

**Authors:** Wenquan Tang, Bin He, YuLin Luo, Xuanchu Duan

**Affiliations:** 1 Department of Ophthalmology, Hunan Children’s Hospital, Changsha, 410007 China; 2 Changsha Aier Eye Hospital, Changsha, 410011 China; 3 Aier School of Ophthalmology, Central South University, Changsha, 410011 China; 4 College of pharmacy, changsha medical university, Changsha, 410011 China

**Keywords:** High myopia, Pathologic myopia, Optic nerve head, Morphology, Peripapillary vessel density

## Abstract

**Purpose:**

To investigate morphological and microcirculation changes of optic nerve head (ONH) in simple high myopia (SHM) and pathologic myopia(PM) to evaluate and identify ONH changes in the development of PM.

**Methods:**

A cross-sectional clinical study was used. Medical records from 193 right eyes of 193 patients with high myopia (HM) were included. Using the Topocon swept source optical coherence tomograph (SS-OCT) and fundus camera to detect the parameters, we have assessed the relative position and size of ONH, tilt and rotation of ONH, angle α (Defined as between retinal temporal arterial vascular arcades was measured from the centre of ONH with 250 pixels’ radius), size and type of peripapillary atrophy (PPA), the thickness of peripapillary retinal nerve fiber layer (PRNFL), peripapillary choriodal thickness (PCT) and peripapillary scleral thickness (PST), and peripapillary vessel density (PVD). In addition, subjects were grouped as SHM and PM according to retinopathy, and the above parameters were compared between the two groups.

**Results:**

Patients were divided into the SHM group (138 eyes) and the PM group (55 eyes). Paramters like older age, higher diopter and longer axial length (AL) of the PM were compared to SHM (t=-3.585, -8.808, -11.409, all P<0.05). There were no differences in the smallest diameter and area of ONH, rotation angle and ratio, or PST (all P>0.05). The angle α in PM was smaller than that in SHM (t = 2.728, P<0.01). The disc-fovea distance (DFD), the largest diameter, tilt index and ratio, PPA area and radian in PM were larger than in SHM (t=-3.962, Z=-2.525, t=-2.229, Z=-4.303, Z=-2.834, all P<0.05). The superior and inferior PRNFLs in PM were smaller than in SHM (t = 4.172, 4.263, all P<0.01). The temporoinferior PRNFL was the opposite (t=-2.421, P<0.01). The average PCT in PM (93.82 ± 29.96 μm) was smaller than in SHM (108.75 ± 30.70 μm) (P<0.05). The PVD in each direction of PM was smaller than that in SHM (t = 6.398, 4.196, 4.971, 3.267, 5.029, 5.653, 4.202, 5.146, 2.090, all P<0.05).

**Conclusion:**

Compared with SHM, the PM patients were older, with higher diopter. Their AL and DFD were longer, the angle α was smaller, the tilt index was more extensive, the PPA area and radian were larger, PCT was generally thinner, and PVD was lower. When the PPA area was bigger than the ONH area, this already indicated the presence of PM. Based on these results, we suggest ophthalmologists and myopia patients pay more attention to ONH’s morphology and microcirculation changes as there is a possibility that microcirculatory changes precede morphologic changes.

## Introduction

Pathologic myopia(PM) is a fundamental cause of irreversible low vision and blindness, leading to irreparable visual impairment. It has a population incidence of 0.9%~3.1% among Asian countries [1]. Typical fundus lesions of PM include fundus tigre, lacquer crack, retinal, choroidal atrophy, choroid neovascular, macular atrophy, posterior scleral staphyloma and retinosis. Although PM is irreversible, it urgently needs to be diagnosed on time, which requires early identification. A previous study found that changes in the optic nerve head (ONH) were the earliest pathological alterations in myopia [2].

Therefore, we hypothesize that the on-time evaluation and identification of early ONH changes in HM will be conducive to the early diagnosis and treatment of PM. Furthermore, before fundus lesions occurred, patients were reasonably treated to reduce vision-threatening complications.

## Methods

### Participants and study design

One hundred ninety-three patients were enrolled in the retrospective study. They were diagnosed with myopia from May 2020 to January 2021 at Changsha Aier Eye Hospital, Central South University, Changsha, China. The study adhered to the Declaration of Helsinki and was approved by the Ethics Committee of Changsha Aier Eye Hospital, Central South University, Changsha, China. In addition, comprehensive examinations including slit-lamp biomicroscopy, mydriatic indirect ophthalmoscopy, intraocular pressure (IOP), axial length (AL) (IOL Master 700; Carl Zeiss Meditec, Germany), ultra-widefield fundus imaging (Optos; Daytona P200T, Nikon, Japan), swept-source optical coherence tomography (SS-OCT) (DRIOCT Triton, Topcon, America), visual acuity and refractive error were conducted for all participants.

The inclusion criteria were: (1) the diopter > -6.00 DS and the AL > 26 mm; (2) IOP ≤ 21 mmHg; (3) SS-OCT acquisition signal ≥ 50. In addition, patients were excluded from the study if they had hypertension, diabetes, systemic connective tissue disorder, any other ophthalmic disease (such as cataract, glaucoma, macular hole, retinal hole, retinal haemorrhage etc.), special abnormal ONH (such as megalodiscs, irregularly shaped discs, extremely small discs etc.), history of ophthalmic trauma and surgery. One hundred ninty-three patients were enrolled in the retrospective study.

According to Meta-PM [3], myopia without fundus lesions and just fundus tigre is considered SHM. PM was diagnosed in patients with diffuse retinal, choroidal atrophy, patchy retinal, choroidal atrophy, macular atrophy, or any combined additional lesions: lacquer cracks, choroidal neovascularization, Fuchs plaque, scleral staphyloma.

### Parameters from Optos and SS-OCT measurements

**Fig. 1:**
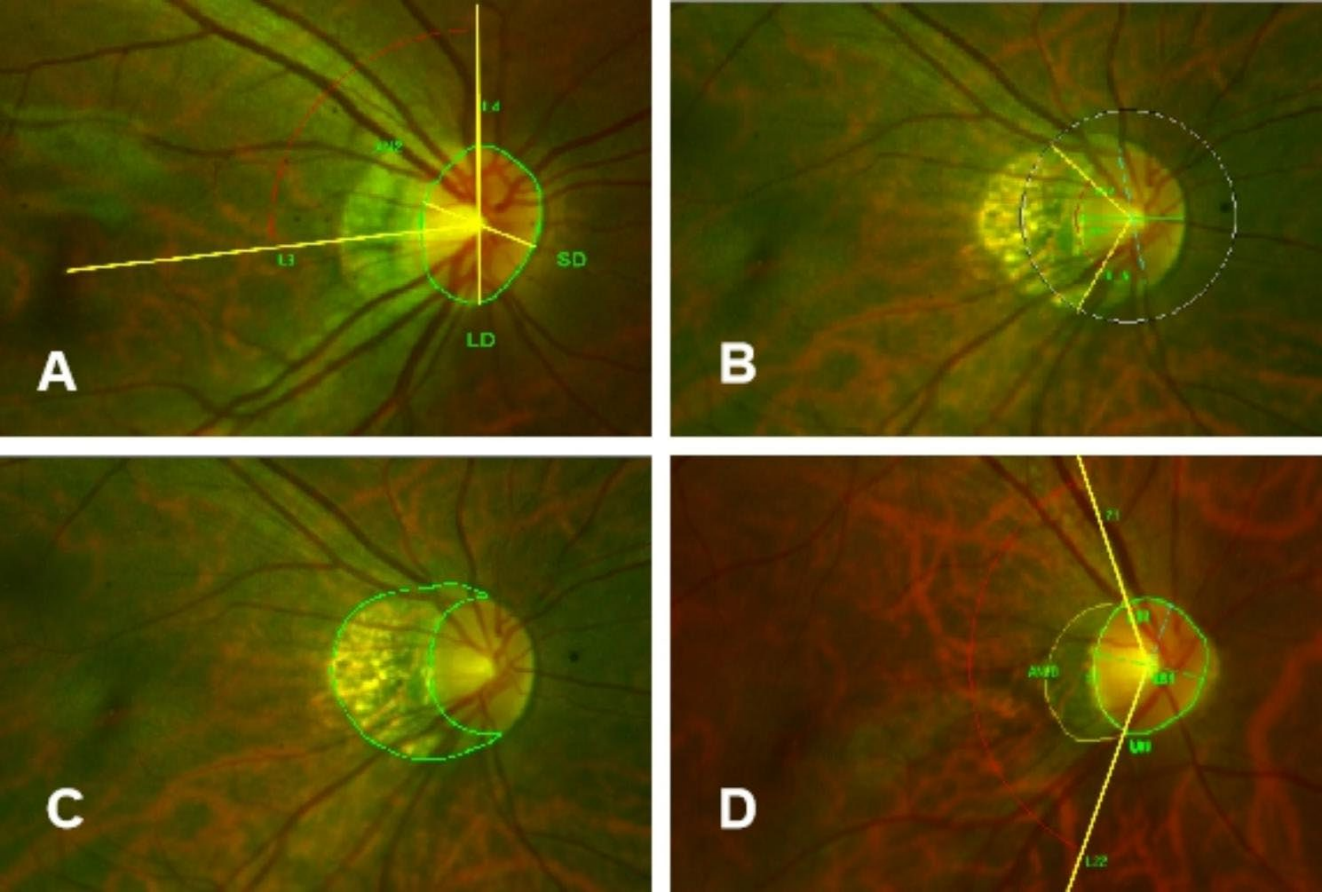
Parameters by Optos. (**A**) The area of ONH was outlined manually. The tilt index was defined as the ratio between the largest diameter (LD) and ONH’s minor diameter (SD). Rotation was measured between the LD and the vertical meridian identified as a vertical line 90°from the horizontal line connecting the fovea and the centre of ONH. DFD was measured between the macular central fovea to the centre of ONH; (**B**) The angle α: A circle (centre: the centre of ONH; radius: 250 pixels) with two intersection points with the upper and lower main temporal retinal arteries was measured for the angle formed by these two intersections and the centre of the circle; (**C**) The area of PPA was outlined manually; (**D**) PPA radian is the radian of PPA centred on the ONH.

We used the Optos to perform digital photography, and the images were exported into the Image-pro Plus (version 6.0) to measure the morphology of ONH. The size, disc-fovea distance (DFD), angle α, tilt, rotation, peripapillary atrophy (PPA) area, and PPA radian were measured from these photographs by the aforementioned retinal ophthalmologists (Fig. 1). The area of PPA (an inner crescent of the chorioretinal atrophy with the visible sclera and choroidal vessels) and ONH were expressed as the total number of pixels using the Image-Pro Plus. The angle α between retinal temporal arterial vascular arcades was measured from the centre of ONH with 250 pixels’ radius [4]. The definitions of tilt and rotation were explained before [5]. Briefly, the tilt was quantified by the tilt index, defined as the ratio between the largest diameter (LD) and the minor diameter (SD) of ONH. ONH was considered a tilt when the tilt index was above 1.3. The rotation was defined as the angle between the long axis and the vertical meridian of ONH. The vertical meridian was defined as a vertical line that passed through the centre of ONH and at 90° from the horizontal line, which connects the fovea and the centre of ONH. The ONH was considered rotated when the rotation angle was more significant than 15°.

**Fig. 2:**
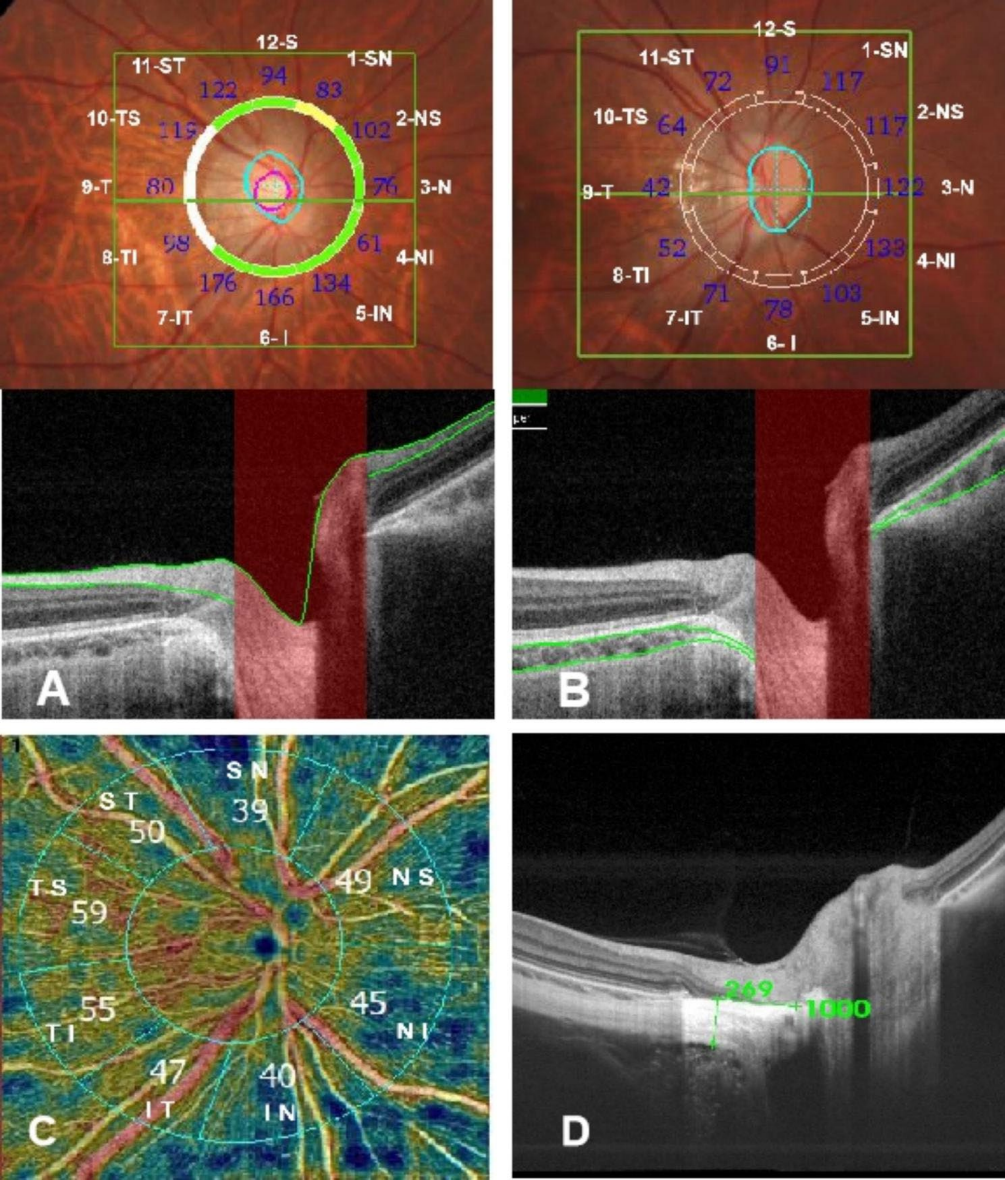
Parameters by SS-OCT. (**A**) peripapillary retinal nerve fiber layer(PRNFL); (**B**) peripapillary choriodal thickness(PCT); (**C**) peripapillary vessel density(PVD); (**D**) peripapillary scleral thickness(PST): We scanned the retina horizontally at 0°in a 12 mm single-line scan mode(centred on the fovea), manually measuring the scleral thickness within 1000 μm from the temporal scleral tube orifice, taking the average of three measurements

We have applied the SS-OCT (centred on the ONH, with a scanning range of 6 × 6 mm), where the system automatically measures the peripapillary retinal nerve fibre layer (PRNFL) thickness, peripapillary choroidal thickness (PCT), peripapillary scleral thickness (PST), and peripapillary vessel density (PVD) (Fig. 2). We divided the ONH into 12 directions (1-superonasal, SN; 2-nasosuperior, NS; 3-nasal, N; 4-nasoinferior, NI; 5-inferonasal, IN; 6-inferior, I; 7-inferotemporal, IT; 8-temporoinferior, TI; 9-temporal, T; 10-temporosuperior, TS; 11-superotemporal, ST; 12-superior, S), Before the SS-OCT image acquisition, we input the examinee’s information (including diopter, corneal curvature and AL) to adjust the magnification factor. The average value was used in the final analysis.

### Statistical analysis

The SPSS22.0 software was used for the statistical analysis of all gathered data. Wherever the data had the normal distribution, it was presented as mean ± standard deviation (X ± SD). The LSD test was applied for the different analyses of the data collected for the two groups of patients. Wherever the data could not meet the normal distribution, it was represented as median (upper quartile, lower quartile), and the non-parametric test (Mann-Whitney (U)) was used for further analysis. The count data were expressed as a percentage (%) and were analyzed with the χ^2^ test. P-values of < 0.05 were considered to be statistically significant.

## Results

### Demographic data and morphological parameters of participants

A total of 193 patients were enrolled in the retrospective study. The right eyes of the patients that met the inclusion and exclusion criteria were analyzed. Among them, 138 eyes were SHM, and 55 eyes were PM. As shown in Table 1, we could not detect any significant differences between the two groups’ SD, area, rotation and avg-PST. Compared with the PM group (30.80 ± 8.88 years), the patients in the SHM group (26.16 ± 5.51 years) were significantly younger (P = 0.010). However, there were significant differences in the proportion of genders between the PM group (26:29) and the SHM group (42:96) (P = 0.027). In addition, the PM group showed longer AL, longer LD, more extended DFD, more considerable SE, more significant tilt ratio, bigger tilt index, bigger PPA and radian, smaller angle α, minor full-peripapillary sclera appearance, thinner PRNFL, thinner PCT, than the SHM group (P < 0.05).

**Table 1:** Demographic data, morphological parameters of SHM and PM.

	SHM(n = 138)	PM(n = 55)	t/Z/χ^2^	P
Age(yrs)	26.16 ± 5.51	30.80 ± 8.88	-3.585	0.010^*^
Gender(male: female)	42:96	26:29	4.886	0.027^*^
AL(mm)	26.62 ± 1.04	28.54 ± 1.48	-8.808	0.000^**^
SE(D)	-8.35 ± 1.26	-12.89 ± 2.84	-11.409	0.000^**^
DFD(Pixel)	430.53 ± 25.09	450.41 ± 33.38	−3.962	0.000^**^
angle α(°)	122.85 ± 22.34	113.77 ± 17.39	2.728	0.007^**^
LD(Pixel)	155.59(145.02,165.46)	162.19(147.14,182.73)	−2.525	0.012^*^
SD(Pixel)	125.02 ± 17.27	131.21 ± 28.79	−1.489	0.141
area(Pixel)	15,077(13291.5,17487.5)	16,173(13582,20187)	−1.450	0.147
tilt index	1.27 ± 0.13	1.32 ± 0.18	−2.229	0.027^*^
tilt ratio(%)	33.33%	49.09%	4.152	0.042^*^
rotation angle (°)	24.64(12.28,47.99)	27.60(15.65,52.47)	−0.838	0.402
rotation ratio (%)	70.29%	76.36%	0.720	0.396
PPA area(Pixel)	6900(3977.25,11757.25)	14,373(5876,30341)	−4.303	0.000^**^
PPA/ONH	0.52 ± 0.04	1.24 ± 0.17	−4.070	0.000^**^
PPA radian(°)	145.45(112.22,179.03)	166.98(121.88,257.71)	−2.834	0.005^*^
Avg-PRNFL(µm)	104.96 ± 10.70	101.14 ± 8.88	2.347	0.020^*^
Avg-PCT(µm)	108.75 ± 30.70	93.82 ± 29.96	3.072	0.002^*^
Avg- PST(µm)	340.44 ± 25.58	335.26 ± 34.11	0.786	0.435
full-peripapillary sclera appearance(%)	64(46.4%)	35(63.6%)	4.689	0.030^*^

SHM = simple high myopia; PM = pathologic myopia; AL = axial length; SE = spherical equivalent; DFD = disc-fovea distance; LD = largest diameter; SD = smallest diameter; PPA = peripapillary atrophy; ONH = optic nerve head; PRNFL = peripapillary retinal nerve fiber layer; PCT = peripapillary choriodal thickness; PST = peripapillary scleral thickness. ^*^P < 0.05; ^**^P < 0.001

### PRNFL parameters between the groups

We have compared the PRNFL parameters among the PM and SHM groups, and the results are displayed in Table 2. The obtained results showed that the measured Avg-PRNFL of the PM group vs. SHM group was 104.96 ± 10.70 μm vs. 101.14 ± 8.88 μm (P = 0.02), SN-PRNFL was 97.56 ± 20.09 μm vs. 91.27 ± 19.03 μm (P = 0.048), I-PRNFL was 122.91 ± 18.58 μm vs. 109.95 ± 20.20 μm (P < 0.001), TI-PRNFL was 88.58 ± 18.52 μm vs. 95.86 ± 19.64 μm (P = 0.016), ST-PRNFL was 131.01 ± 20.60 μm vs. 122.23 ± 23.80 μm (P = 0.011) and S-PRNFL was 114.21 ± 17.90 μm vs. 102.53 ± 16.68 μm (P < 0.001), respectively. Nevertheless, there were no significant differences in the nasosuperior (NS), nasal (N), nasoinferior (NI), inferotemporal (IT), temporal (T), and temporosuperior (TS).

**Table 2:** PRNFL parameters of SHM and PM.

	SHM(n = 138)	PM(n = 55)	t	P
Avg-PRNFL(µm)	104.96 ± 10.70	101.14 ± 8.88	2.347	0.020^*^
SN of PRNFL(µm)	97.56 ± 20.09	91.27 ± 19.03	1.991	0.048^*^
NS of PRNFL(µm)	65.93 ± 16.42	62.66 ± 13.54	1.312	0.191
N of PRNFL(µm)	62.73 ± 15.05	61.59 ± 12.67	0.497	0.620
NI of PRNFL(µm)	59.53 ± 15.89	60.42 ± 14.42	-0.361	0.718
IN of PRNFL(µm)	94.92 ± 22.74	87.29 ± 22.30	2.115	0.036
I of PRNFL(µm)	122.91 ± 18.58	109.95 ± 20.20	4.263	0.000^**^
IT of PRNFL(µm)	151.04 ± 21.57	143.67 ± 28.36	1.739	0.086
TI of PRNFL(µm)	88.58 ± 18.52	95.86 ± 19.64	-2.421	0.016^*^
T of PRNFL(µm)	97.93 ± 17.75	103.51 ± 20.48	-1.884	0.061
TS of PRNFL(µm)	107.25 ± 19.96	110 ± 25.83	-0.793	0.429
ST of PRNFL(µm)	131.01 ± 20.60	122.23 ± 23.80	2.552	0.011^*^
S of PRNFL(µm)	114.21 ± 17.90	102.53 ± 16.68	4.172	0.000^**^

SHM = simple high myopia, PM = pathologic myopia, SN = superonasal, NS = nasosuperior, N = nasal, NI = nasoinferior, IN = inferonasal, I = inferior, IT = inferotemporal, TI = temporoinferior, T = temporal, TS = temporosuperior, ST = superotemporal, S = superior, PRNFL = peripapillary retinal nerve fiber layer. ^*^P < 0.05; ^**^P < 0.001

### PCT parameters between groups

The comparison among the PCT parameters between the patients’ groups is displayed in Table 3. Their measurements showed that Avg-PCT of the PM group vs. SHM group was 108.75 ± 30.70 μm vs. 93.82 ± 29.96 μm (P = 0.002), SN-PCT was 130.76 ± 38.81 μm vs. 110.27 ± 38.43 μm (P = 0.001), NS-PCT was 126.66 ± 40.96 μm vs. 106.61 ± 35.56 μm, (P = 0.001), N-PCT was 131.08 ± 39.93 μm vs. 110.64 ± 28.39 μm (P < 0.001), NI-PCT was 126.52 ± 42.66 μm vs. 102.87 ± 30.40 μm (P < 0.001), IN-PCT was 92.07 ± 29.67 μm vs. 110.01 ± 40.27 μm (P = 0.001), I-PCT was 99.41 ± 39.91 μm vs. 86.02 ± 28.86 μm (P = 0.011) and ST-PCT was 116.43 ± 40.71 μm vs. 99.36 ± 39.41 μm (P = 0.009), respectively. These data show that these parameters were significantly thinner in the PM group than in the other. Nevertheless, there were no significant differences in the other parameters like IT, IT, T, TS, and S.

**Table 3:** PCT parameters of SHM and PM.

	SHM(n = 138)	PM(n = 55)	t	P
Avg- PCT(µm)	108.75 ± 30.70	93.82 ± 29.96	3.072	0.002^*^
SN of PCT(µm)	130.76 ± 38.81	110.27 ± 38.43	3.320	0.001^**^
NS of PCT(µm)	126.66 ± 40.96	106.61 ± 35.56	3.384	0.001^**^
N of PCT(µm)	131.08 ± 39.93	110.64 ± 28.39	3.993	0.000^**^
NI of PCT(µm)	126.52 ± 42.66	102.87 ± 30.40	4.318	0.000^**^
IN of PCT(µm)	110.01 ± 40.27	92.07 ± 29.67	3.404	0.001^**^
I of PCT(µm)	99.41 ± 39.91	86.02 ± 28.86	2.593	0.011^*^
IT of PCT(µm)	87.86 ± 38.99	80.05 ± 30.44	1.330	0.185
TI of PCT(µm)	77.24 ± 36.45	73.13 ± 35.89	0.710	0.478
T of PCT(µm)	79.42 ± 63.87	67.42 ± 40.40	1.293	0.198
TS of PCT(µm)	89.22 ± 36.13	78.60 ± 38.46	1.810	0.072
ST of PCT(µm)	116.43 ± 40.71	99.36 ± 39.41	2.652	0.009^*^
S of PCT(µm)	130.42 ± 39.37	118.78 ± 40.34	1.841	0.067

SHM = simple high myopia, PM = pathologic myopia, SN = superonasal, NS = nasosuperior, N = nasal, NI = nasoinferior, IN = inferonasal, I = inferior, IT = inferotemporal, TI = temporoinferior, T = temporal, TS = temporosuperior, ST = superotemporal, S = superior, PCT = peripapillary choriodal thickness. ^*^P < 0.05; ^**^P < 0.001

### PVD parameters between the groups

The comparison between the PVD parameters between the two groups is shown in Table 4. Our results showed that the significant differences between the PVD parameters between the two groups were in SN, NS, NI, IN, IT, TI, and TS(t = 6.398, 4.196, 4.971, 3.267, 5.029, 5.653, 4.202, 5.146, all P<0.001). For example, the measured Avg-PVD was 50.58 ± 2.99% in the PM group vs. 47.59 ± 2.78% in the SHM group (P < 0.001).

**Table 4:** PVD parameters of SHM and PM.

	SHM(n = 138)	PM(n = 55)	t	P
Avg-PVD(%)	50.58 ± 2.99	47.59 ± 2.78	6.398	0.000^**^
SN of PVD (%)	49.07 ± 4.11	46.14 ± 4.98	4.196	0.000^**^
NS of PVD (%)	44.97 ± 5.16	40.66 ± 6.10	4.971	0.000^**^
NI of PVD (%)	44.11 ± 5.88	41.01 ± 6.17	3.267	0.001^**^
IN of PVD (%)	49.01 ± 5.14	44.62 ± 6.25	5.029	0.000^**^
IT of PVD (%)	57.16 ± 4.49	53.09 ± 4.56	5.653	0.000^**^
TI of PVD (%)	54.93 ± 5.41	50.83 ± 7.60	4.202	0.000^**^
TS of PVD (%)	56.27 ± 5.21	51.52 ± 7.07	5.146	0.000^**^
ST of PVD (%)	54.38 ± 4.43	52.82 ± 5.29	2.090	0.038^*^

SHM = simple high myopia, PM = pathologic myopia, SN = superonasal, NS = nasosuperior, NI = nasoinferior, IN = inferonasal, IT = inferotemporal, TI = temporoinferior, TS = temporosuperior, ST = superotemporal, PVD = peripapillary vessel density. *P < 0.05; **P < 0.001

### Comparison of morphology and microcirculation between SHM and PM groups

**Fig. 3:**
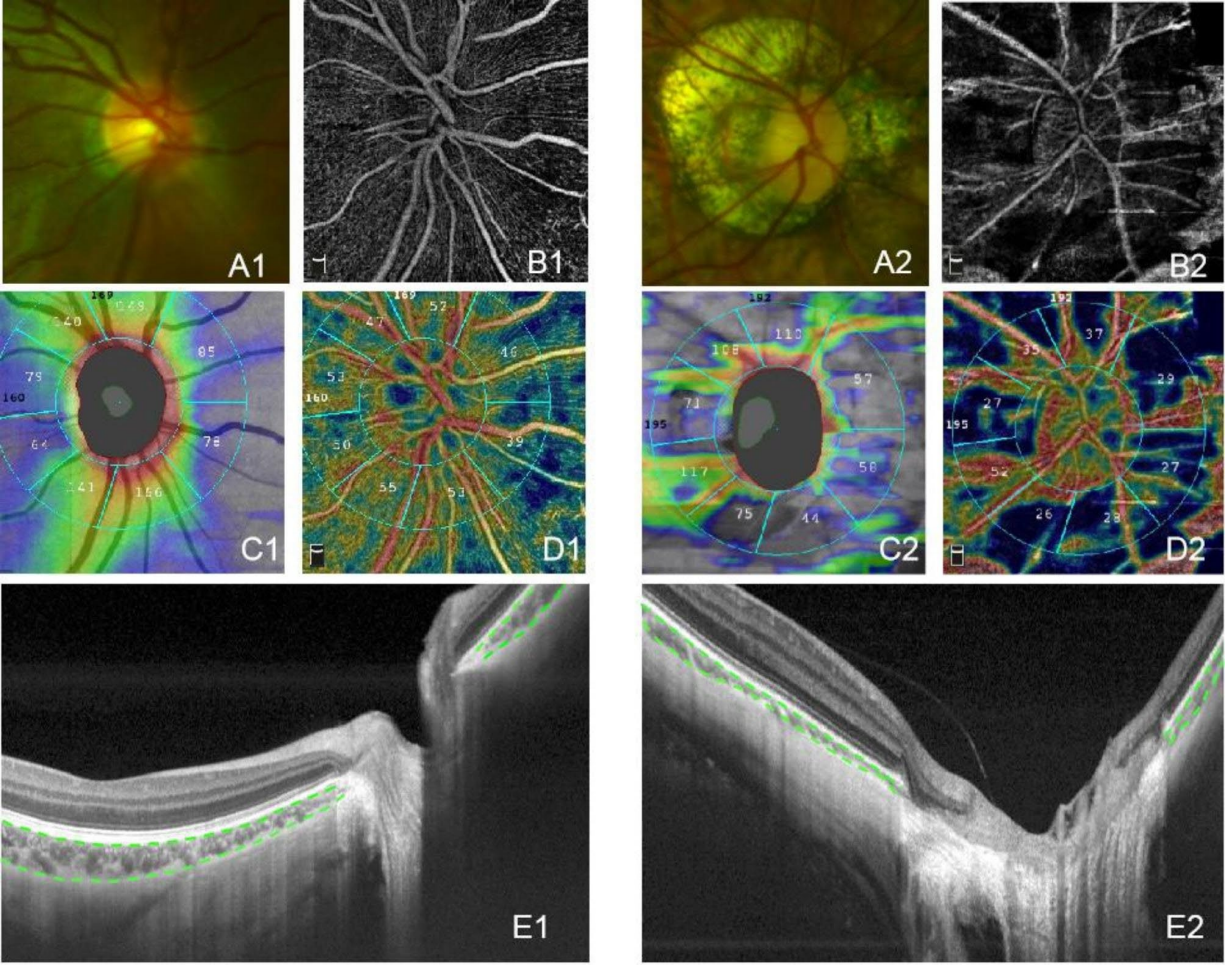
OCT images of SHM and PM. A is the morphological image of fundus photography; B and D are the PVD image of OCT; C is the PRNFL image of OCT; E is the structure image of OCT.

The comparison of morphology and microcirculation of the eyes of the patients’ in the compared groups are shown in Fig. 3. A1 shows an ONH with reddish, oval, temporal type PPA, belonging to SHM; A2 shows an ONH with yellowish, oval, toroidal type PPA, belonging to PM; B and D show that the microvascular density of PM is reduced, accompanied by a patch defect area. C shows significant changes in PRFNL distribution in PM compared with SHM. E shows no changes in PST in PM compared with SHM, but the full-peripapillary sclera appearance is enhanced, and PCT is thinner.

## Discussion

At present, the clinical definition of PM is not clear. Therefore, it is necessary to discover the unique features of PM by measuring parameters that describe the morphology and microcirculation changes of the optic nerve head between SHM and PM. Hence, the primary purpose of this study is to compare the difference in ONH between SHM and PM to find the measurable indicators of morphological and microcirculatory parameters related to ONH.

### The relative position of ONH

Previous studies have shown that axial myopia mainly depends on the posterior eyeball enlargement, and the closer the posterior pole is, the pathology is more prominent [6]. To assess the extent of expansion of the posterior eyeball, we measured the DFD and the angle α. DFD describes the relative position of ONH and fovea. We found that the DFD in the PM group was longer than in the SHM group. In another prospective study [7], it was found that DFD increased from 4.98 ± 0.3 mm to 6.11 ± 0.5 mm in adolescents with an increasing AL. As a characteristic indicator of the posterior polar morphological changes of HM, the angle α is considered an essential reference for the nasal displacement of ONH [8]. We found that the angle α of SHM (122.85 ± 22.34) was more prominent than in the PM group (113.77 ± 17.39). Compared with the SHM, the distance between the ONH and the fovea in the PM group was further widened. The upper and below retinal vessels were pulled to cause the reduction of the vascular clamp [20]. In a prospective observational study, Lee [8] found that with the axial elongation of myopia, the central vascular trunk of the retina was dragged nasally, considering that nasally passive traction of the lamina cribiosa was the main reason. Therefore, changes in the DFD and the angle α can be explained by the expansion of the posterior eyeball in myopia. The closer the posterior pole is, the more apparent the pathology is. Therefore, it is once again proved that the posterior eyeball enlargement and the peripapillary tissue pulling displacement are progressive aggravations in PM.

### The shape and size of ONH

Normal ONH is round or oval with an average diameter of 1.5 mm. ONH is the access of the optic nerve and retinal vessels in the eye, consisting of nerves, vessels and bindweb. The morphology changes of ONH are often manifested as tilt and PPA in HM. Results show no significant difference between the area and SD of ONH in the SHM group and PM group, while LD and tilt index of ONH were significantly different. In contrast, the tilt ratio increased in PM. The severe tilt and distorted deformation adversely impacted the optic nerve, resulting in vision decline [9]. This indicated that the identifiable or quantitative parameters should be obtained from the tilt and deformation of ONH.

### The size and type of peripapillary atrophy (PPA)

PPA is attributed to photoreceptor reduction, RPE and choroid capillary loss, Bruch membrane displacement or defects. As a characteristic change of myopia, the expansion of PPA indicates the progression of myopia. During 20 years of prospective studies on fundus in children with HM, Yokoi [10] found that 87% of adults with PM had diffuse choroid retinal atrophy in childhood, suggesting that adult patients with PM already showed a fundus appearance different from the fundus appearance in children with benign myopia. Meanwhile, in childhood, the sign of peripapillary diffuse chorioretinal atrophy is an indicator or biomarker for more advanced myopic chorioretinal atrophy in later life. Our study showed that the PPA area and radian of the PM group were significantly more significant than the SHM. As the image J software did not calculate the actual area size due to image analysis, we introduced the ratio of PPA area / ONH area (PPA/ONH) to estimate the size of the PPA. The PPA/ONH of the PM group was 1.24 ± 0.17, significantly greater than 0.52 ± 0.04 in the SHM group. It indicated that when the PPA area was more significant than the ONH area, this already indicated the presence of PM.

### The thickness of the peripapillary tissue

ONH is seamlessly connected to the RNFL, choroid, and sclera, which plays a crucial role in morphology changes. PRNFL mainly shows changes in thickness and distribution in HM. Our results showed that the average thickness of PRNFL in the PM group was lower than that in the SHM group, significantly inferior and superior; However, the TI-PRNFL of the PM group was slightly higher than that of the SHM group. Analogously, while studying the changes in displacement and morphology of ONH, Tan [11] found that the angle between the superior and inferior temporal RNFL bundles decreased by 3.3° for every 1 mm increase in AL. A reasonable explanation was that the retina pulls along with the ONH to the nasal side in axial myopia, and the inferior and superior RNFL moved closer toward the temporal side. Clinically, the peak RNFL position is often shifted to the temporal side, leading to relative thickening.

The choroid, as vascular tissue, lies between the scleral and retina. Compared with ordinary people, in anatomy and OCT, the choroid was atrophic and thin in HM [12]. Moreover, our study further demonstrated that the choroid of PM (93.82 ± 29.96 μm) was thinner compared with SHM (108.75 ± 30.70 μm). Subregional measurements showed that nasal and superior PCT were thicker than the inferior and temporal PCT, where the temporal side was the thinnest. This heterogeneity of altered choroid may be an intrinsic reason why myopia-associated fundus lesions often occur on ONH’s temporal side [13]. Choroids support not only outer retinal nutrition but also release multiple vascular factors to regulate scleral growth [14]. Studies have found that a compensatory increase in retinal vessel oxygen saturation was observed in moderate myopia, which might be related to insufficient choroid blood supply to the outer retina and increased oxygen consumption. But this compensatory effect disappears immediately when HM occurs, making the retina more prone to pathological changes. Meanwhile, Liu [14] found that the choroid is involved in transmitting biological signals from the retina to the sclera and regulates scleral remodelling and staphyloma. These findings suggest more attention to be paid to choroids to assess the progression of PM.

As the outermost layer of the eyeball, the sclera plays an essential role in stabilizing intraocular pressure, protecting the intraocular structure and maintaining normal morphology. The morphology and structure of the sclera were shown to change constantly, together with its biochemical and biomechanical properties, along with AL, including the phenotype of the scleral fibroblasts and the composition of the extracellular matrix [15]. Whether the posterior scleral bound is detected by SS-OCT is closely related to the choroidal thickness and the Bruch membrane atrophy, so the entire sclera appearance reflects the pathologic status of the eyeball. Ohno-Matsui used the SS-OCT to find that 57% of the eyes showed the whole scleral structure, and Tenon’s capsule and orbital fat tissue could also be shown in some eyes [16]. In this study, the rate of full-peripapillary sclera appearance in whole subjects reached 51.3%, and the rate in PM (63.6%) was significantly higher than in SHM (46.4%). This also meant that scleral tissue atrophy was much higher in the PM than in SHM. However, our studies obtained no comparable parameters by scleral thickness, suggesting that PST is not a reliable PM indicator.

We further speculate that the choroidal changes may precede the RNFL and sclera by affecting the blood supply of the two tissues, stimulating myopia toward HM, and ultimately resulting in PM. These initial insights may provide clues and a basis for further investigation of histology in HM.

### The microcirculation around the ONH

In our study, the PVD was lower in the PM group compared with the SHM. Similarly, another study showed that HM had less peripapillary retinal blood flow index and vessel density (VD) than emmetropia, suggesting decreased peripapillary microcirculation perfusion [17]. We also found that the PVD in PM was lower in each direction than in SHM, and the VD in the temporal direction was higher than in the nasal direction in both groups. This result suggested that the blood flow was preferentially perfused in the bow-shaped nerve fibre region to meet the metabolic energy needs of RNFL, just as no significant VD reduction was found in the macula of HM. Such relative retention of retinal blood flow perfusion in the vital area ensured normal visual function. At the same time, we found that the PCT in the nasal direction was thicker than that in the temporal direction, quite the opposite of the results of retinal blood perfusion. The reason might be due to the anatomical characteristics of the ONH, and the complex blood circulation, mainly provided by the retinal and choroidal circulation.

Meanwhile, the Zinn-Haller provided the main blood supply to the lamina cribiosa of ONH. Ishida [18] found that the distance between the Zinn-Haller and the boundary of ONH increased significantly with AL. On the one hand, the Zinn-Haller might play a key role in maintaining blood perfusion stabilization in the ONH. On the other hand, the progressive tilt and rotation of ONH were often accompanied by abnormal and slow choroidal circulation and terminal artery occlusion in HM. This suggested that it was easy for the circulation disorder in ONH to occur. However, the mechanism of reduced retinal and choroidal perfusion is unclear. It is often believed that hyper expansion causes the retina and choroidal thinning in HM, and these thinning tissues might reduce oxygen demand and, thus, blood perfusion [19].

We acknowledge several limitations associated with our study. First, this is a retrospective study. Hence, the longitudinal data were unavailable. Thus, it might not reflect the true representative of the population. Furthermore, due to the requirements of SS-OCT scan quality, most patients with PM were excluded from the study, allowing a selection bias. Last, the magnification effect of images might affect the results of OCT measurements (e.g. vessel density and area). Still, the current formula for amplification correction was not uniform, and re-measurements were separated from the OCT measure system, which might bring new deviations. However, the range of 4.5 × 4.5 mm scans used in this study was small enough that the effect of the amplification effect was not significant.

## Conclusion

PM is closely linked to the reduction of choroidal perfusion and structural changes of ONH. Compared with SHM, the PM patients were older, with higher diopter. As a result, their AL and DFD were longer, the angle α was smaller, the tilt index was more extensive, the PPA area and radian were larger, PCT was generally thinner, and PVD was lower. Unfortunately, it was impossible to regain normal vision once PM-related complications occurred. Therefore, we suggest ophthalmologists and myopia patients pay more attention to ONH’s morphology and microcirculation changes as there is a possibility that microcirculatory changes precede morphologic changes. However, this needs to be determined by further studies.

## Data Availability

The data materials were obtained from reasonable request to the corresponding author. All data and materials were unpublished.

## Abbreviations

AL: Axial length
DFD: Disc-fovea distance
ONH: Optic nerve head
PM: Pathologic myopia
HM: High myopia
PPA: Peripapillary atrophy
PRNFL: Peripapillary retinal nerve fiber layer
PCT: Peripapillary choroidal thickness
PST: Peripapillary scleral thickness
PVD: Peripapillary vessel density
SS-OCT: Swept source optical coherence tomography
SHM: Simple high myopia

## References

[1] Wong YL, Saw SM (2016). Epidemiology of pathologic myopia in Asia and Worldwide[J]. Asia Pac J Ophthalmol(Phila).

[2] Samarawickrama C, Mitchell P, Tong L (2011). Myopia-related optic disc and retinal changes in adolescent children from singapore[J]. Ophthalmology.

[3] Ohno-Matsui K (2016). Pathologic Myopia[J]. Asia Pac J Ophthalmol (Phila).

[4] Nie F, Ouyang J,Tang W et al. Posterior staphyloma is associated with the microvasculature and microstructure of myopic eyes. Graefes Arch Clin Exp Ophthalmol, 2021, undefined: undefined. 10.1007/s00417-020-05057-0. 10.1007/s00417-020-05057-0 PMC8352845 33404680

[5] Sung MS, Kang YS, Heo H (2016). Characteristics of Optic Disc Rotation in myopic Eyes[J]. Ophthalmology.

[6] Vurgese S, Panda-Jonas S, Jonas JB (2012). Scleral thickness in human eyes[J]. PLoS One.

[7] Guo Y, Liu LJ, Tang P (2018). Optic disc-fovea distance and myopia progression in school children: the Beijing Children Eye Study[J]. Acta Ophthalmol.

[8] Lee KM, Choung HK, Kim M (2018). Positional change of optic nerve head vasculature during axial elongation as evidence of lamina cribrosa shifting: Boramae myopia cohort study report 2[J]. Ophthalmology.

[9] Pichi F, Romano S, Villani E (2014). Spectral-domain optical coherence tomography findings in pediatric tilted disc syndrome[J]. Graefes Arch Clin Exp Ophthalmol.

[10] Yokoi T, Jonas JB, Shimada N (2016). Peripapillary diffuse Chorioretinal Atrophy in Children as a sign of eventual pathologic myopia in Adults[J]. Ophthalmology.

[11] Tan N, Sng C, Ang M (2019). Myopic optic disc changes and its role in glaucoma[J]. Curr Opin Ophthalmol.

[12] Song A, Hou X, Zhuo J (2020). Peripapillary choroidal thickness in eyes with high myopia[J]. J Int Med Res.

[13] Deng J, Li X, Jin J (2018). Distribution pattern of Choroidal Thickness at the posterior Pole in Chinese Children with Myopia. Invest Ophthalmol Vis Sci.

[14] Liu X, He X, Yin Y (2019). Retinal oxygen saturation in 1461 healthy children aged 7–19 and its associated factors[J]. Acta Ophthalmol.

[15] McBrien NA, Gentle A (2003). Role of the sclera in the development and pathological complications of myopia[J]. Prog Retin Eye Res.

[16] Ohno-Matsui K, Akiba M, Modegi T (2012). Association between shape of the sclera and myopic retinochoroidal lesions in patients with pathologic myopia[J]. Invest Ophthalmol Vis Sci.

[17] Wang X, Kong X, Jiang C (2016). Is the peripapillary retinal perfusion related to myopia in healthy eyes? A prospective comparative study. BMJ Open.

[18] Ishida T, Jonas JB, Ishii M (2017). Peripapillary arterial ring of zinn-haller in highly myopic eyes as detected by optical coherence tomography angiography[J]. Retina.

[19] Ohno-Matsui K, Jonas JB (2019). Posterior staphyloma in pathologic myopia[J]. Prog Retin Eye Res.

[20] Jonas JB, Weber P, Nagaoka N (2018). Temporal vascular arcade width and angle in high axial myopia[J]. Retina.

